# Association between the APOE *ε*4 Allele and Late-Onset Alzheimer's Disease in an Ecuadorian Mestizo Population

**DOI:** 10.1155/2017/1059678

**Published:** 2017-12-04

**Authors:** Stefany Montufar, Cristian Calero, Rodrigo Vinueza, Patricio Correa, Andrea Carrera-Gonzalez, Franklin Villegas, Germania Moreta, Rosario Paredes

**Affiliations:** ^1^Hospital de Especialidades FFAA, Quito, Pichincha, Ecuador; ^2^Hospital Carlos Andrade Marín, Quito, Pichincha, Ecuador; ^3^Hospital Metropolitano, Quito, Pichincha, Ecuador

## Abstract

Alzheimer's disease (AD) is the most common neurodegenerative disease. It has two main pathological hallmarks: amyloid plaques and neurofibrillary tangles. The APOE *ε*4 allele has been recognized as the strongest genetic risk factor for late-onset Alzheimer's disease (LOAD) in several populations worldwide, yet the risk varies by region and ethnicity. The aims of this study were to describe APOE allele and genotype frequencies and examine the relationship between the APOE *ε*4 allele and LOAD risk in an Ecuadorian Mestizo population. We carried out a case-control study comprising 56 individuals clinically diagnosed with probable AD (≥65 years of age) and 58 unrelated healthy control subjects (≥65 years of age). Genotyping was performed using the real-time PCR method. Our data showed that allelic and genotypic frequencies follow the trends observed in most worldwide populations. We also found a high-risk association between APOE *ε*4 allele carriers and LOAD (OR = 7.286; 95% CI = 2.824–18.799; *p* < 0.001). Therefore, we concluded that APOE *ε*4 must be considered an important genetic risk factor for LOAD in the Ecuadorian Mestizo population. Additionally, we suggest that in mixed populations the effects of admixture and ethnic identity should be differentiated when evaluating genetic contributions to Alzheimer's disease risk.

## 1. Introduction

Alzheimer's disease (AD) has emerged as the most prevalent form of late-life mental failure in humans, accounting for about 60–80% of dementia cases [1]. Alzheimer's Disease International (ADI) estimated that 46.8 million people worldwide were living with dementia in 2015 and predicted that this figure would reach 131.5 million in 2050 [2]. In Latin America, due to demographic and health transitions as well as low socioeconomic levels, it is expected that the number of people with dementia will rise from 7.8 million in 2013 to over 27 million by 2050 [3, 4]. The analysis of eight population studies conducted in Brazil, Cuba, Chile, Peru, and Venezuela showed that the global prevalence of dementia in Latin America has reached 7.1%, with AD being the most frequent type [5]. In Ecuador, there are no official data of prevalence or incidence of dementia.

AD is a progressive and irreversible neurodegenerative disorder characterized by a decline in memory, language, problem-solving, and other cognitive skills that affect an individual's ability to perform daily activities [1]. The key neuropathological features of AD are extracellular plaques composed of *β*-amyloid peptides (A*β*) and intracellular neurofibrillary tangles (NFTs), consisting of hyperphosphorylated tau proteins [6, 7]. Other neuropathological changes are synapse and neuronal loss, cerebral atrophy, gliosis, white matter degeneration, granulovacuolar degeneration, cerebral amyloid angiopathy (CAA), and other protein aggregates (TAR DNA-binding protein 43-immunoreactive inclusions, Lewy bodies, and actin-immunoreactive Hirano bodies) [7].

Based on the age of onset, AD is classified into two types: early-onset AD (EOAD, onset < 65 years of age) accounting for 1–5% of all cases and late-onset AD (LOAD, onset ≥ 65 years of age) accounting for >95% of them [8]. Three dominant inherited mutations in the genes amyloid precursor protein (APP), presenilin-1 (PS1), and presenilin-2 (PS2), which increase the production of amyloid-beta (A*β*) peptides, have been linked to EOAD [9, 10]. Conversely, LOAD has been identified to be more complex involving multiple susceptibility genes and environmental factors [11]. Genetically, the APOE *ε*4 allele has been recognized as the strongest risk factor for LOAD [12–14].

Apolipoprotein E (APOE) is a glycoprotein of 299 amino acids with an estimated molecular mass of ~34 kDa that is involved in cholesterol transport and encoded by the APOE gene located in the long arm of the chromosome 19q13.2 [15]. The human APOE gene exists as three polymorphic alleles—*ε*2, *ε*3, and *ε*4—which differ by cysteine and arginine amino acids at residues 112 and 158, with *ε*2 (cys112, cys158), *ε*3 (cys112, arg158), and *ε*4 (arg112, arg158) [16].

The APOE *ε*4 allele has been related to earlier onset as well as higher risk for AD, and individuals with two copies of the APOE *ε*4 allele have a higher risk and earlier onset than heterozygous subjects [17]. Several studies have suggested mechanisms that could explain APOE *ε*4 allele's contribution to the pathogenesis of AD. These include the modulation of the deposition and clearance of A*β*, formation of plaques, impairment of the antioxidative defense system, dysregulation of the neuronal signaling pathways, disruption of cytoskeletal structure and function, and increased phosphorylation of tau with formation of neurofibrillary tangles [18].

Few APOE studies have been conducted in Latin America, and in Ecuador only two previous studies have investigated that gene. The first study evaluated frequencies of APOE genotypes and alleles in a population of Cayapas from Esmeraldas (*n* = 96) [19]. However, even though Cayapa Indians are part of the indigenous population living in Ecuador, Mestizos are the most numerous ethnic group accounting for approximately 60% of the total population. Ecuadorian Mestizos can be considered a trihybrid population resulting from the admixture of Amerindian, European, and African populations, having estimated average proportions of ~73, ~19, and ~8% in autosomal cells, respectively [20]. The second study evaluated the association between APOE *ε*4 and AD in a sample consisting of healthy (*n* = 39) and affected (*n* = 39) individuals from different provinces of Ecuador. Nevertheless, the ethnic composition was not detailed and no significant association was found in that study probably due to the limited sample size [21].

Therefore, the aims of this study were to describe APOE genotype and allele frequencies in a control-case study and to determine the relationship between APOE *ε*4 allele carriers and the risk of developing LOAD in an Ecuadorian Mestizo population.

## 2. Materials and Methods

### 2.1. Study Population

Subjects were recruited from the Hospital Carlos Andrade Marín in Quito, Ecuador, from September 2014 until May 2015. All participants were ethnic Mestizo Ecuadorians aged 65 and older.

The case subjects consisted of 56 unrelated patients with a clinical diagnosis of probable AD established by a neurologist according to the National Institute on Aging and the Alzheimer's Association (NIA-AA) criteria [37]. All the patients were subjected to the following procedures: (1) a neurological examination, which included a physical examination and a review of medical history; (2) a computerized axial tomography (CAT), to confirm hippocampal atrophy and exclude hydrocephalus, cerebrovascular disorders, or intracranial mass; (3) a Mini-Mental State Examination (MMSE) test score of 24 or less; (4) laboratory tests (blood count, blood chemistry, alanine transaminase (ALT), aspartate transaminase (AST), vitamin B12 level, folic acid level, and thyroid-stimulating hormone (TSH)) to exclude other diseases than AD that potentially would cause symptoms of dementia; (5) an exclusion of secondary causes of dementia such as depressive disorder, bipolar disorder, schizophrenia, substance use disorder, mental retardation, history of traumatic brain injury, or another neurologic disease.

The control subjects consisted of 58 healthy unrelated volunteers with no signs of psychiatric or neurological impairment, based on clinical examinations which included an MMSE score of 27 or higher.

All procedures were carried out with informed and signed consent from patients or suitable proxies and healthy volunteers. This study was approved by the Central University Bioethics Committee (UCE-COBI) and was performed in accordance with the Ethical Standards of the Declaration of Helsinki.

### 2.2. Genotyping

Genomic DNA extraction was carried out from peripheral blood samples using the* High Pure PCR Template Preparation Kit *(*Roche Diagnostics GmbH*, Mannheim, Germany) in accordance with the manufacturer's protocol.

Real-time PCR (RT-PCR) and genotyping were performed on the LightCycler 2.0 platform, software version 4.1, using the fluorescent resonance energy transfer (FRET) technique (*Roche Diagnostics GmbH*, Mannheim, Germany) with predesigned primers and probes from the* LightMix*®* ApoE C112R R158C kit *(TIB MOLBIOL GmbH, Berlin, Germany).

### 2.3. Statistical Analyses

Deviation from Hardy-Weinberg equilibrium (HWE) of APOE genotype and alleles was calculated with the Pearson chi-square statistical test in both the case and the control populations.

Statistical analyses were performed using the SPSS software, version 22 (IBM-SPSS Inc., Chicago, IL). Categorical variables were described through frequencies and percentages, and quantitative variables were described using means and standard deviations.

Variables were compared between the case and control groups using the *t*-test for quantitative variables and chi-square test with the Yates correction applied or Fisher's exact test for categorical variables. The statistical significance was set at 0.05 for each analysis. Odds ratio (OR) and 95% confidence intervals (CI) were calculated to determine the strength of association between APOE *ε*4 carriers and the risk of developing LOAD.

## 3. Results

### 3.1. Demographic Description

A total of 114 participants were recruited, including 56 LOAD patients (mean age: 78.04 ± 6.32; 53.6% females) and 58 healthy controls (mean age: 76.62 ± 7.07; 53.4% females). Demographic and clinical data are summarized in Table 1. There were no statistical differences in gender (*p* = 1.000), age (*p* = 0.263), smoking (*p* = 0.309), hypertension (*p* = 0.459), diabetes (*p* = 0.942), and years of education (*p* = 0.896) between the two groups. As expected, the MMSE score in LOAD patients was significantly lower when compared with the control subjects' score (*p* < 0.001).

### 3.2. Allele and Genotype Frequency Distributions of APOE Polymorphisms

Analysis of APOE allele frequency data revealed that the case and control populations were in Hardy-Weinberg equilibrium. The distributions of each allele and genotype frequency are shown in Table 2.

APOE *ε*4 allele frequencies were greater for patients with LOAD (33.9%) than for control subjects (6.0%). Furthermore, the statistical analysis showed that being a carrier of at least one APOE *ε*4 allele seems to be a significant risk factor for LOAD (OR = 7.286; 95% CI = 2.824–18.799; *p* < 0.001) (Table 3).

## 4. Discussion

Alzheimer's disease represents one of the greatest global health problems of this century. Not only is it responsible for several deaths, but also it contributes to the increase in cases of poor health and disability [1]. Therefore, AD has become an epidemic affecting individuals and public health systems and, as yet, its current and future impacts have been underestimated [38].

Although associations between the APOE *ε*4 allele and AD risk have been replicated in several populations, the frequencies of the three APOE alleles and the association of APOE *ε*4 exposure with AD are highly variable among different populations [22, 39–41]. Additionally, previously published studies indicate that ethnicity, genetic, and environmental factors all contribute to AD risk [11, 42]. Thus, it is important to study the nature of this association in the specific population of interest.

The present study describes the APOE allele and genotype frequencies of 56 LOAD patients and 58 healthy control individuals in a Mestizo population from Ecuador and their association with LOAD. This is the first report that shows a significant risk association between APOE *ε*4 carriers and LOAD in an Ecuadorian population.

The distribution observed in the healthy control individuals showed that the most frequent APOE allele was *ε*3 (89.7%), followed by *ε*4 (6.0%) and *ε*2 (4.3%). Regarding genotypes, the *ε*3*ε*3 genotype has the most common distribution (81.0%), followed by *ε*3*ε*4 (10.3%), *ε*2*ε*3 (6.3%), and *ε*2*ε*4 (1.7%). These allele and genotype frequencies maintain the trends described in most healthy control populations worldwide such as Caucasians, African Americans, Hispanics, and Japanese [22, 23]. Compared with other Latin American countries, the trends observed are consistent with those previously reported in samples from Ecuadorian and Peruvian Mestizo populations [21, 43] and with South American native populations from Argentina, Brazil, and Paraguay [44]. However, our findings differ from those observed in the Ecuadorian Cayapa population whose trend was *ε*3*ε*3 > *ε*3*ε*4 > *ε*4*ε*4 [19]. This difference could be attributed to the fact that Cayapas are an ethnic homogeneous indigenous population that has adapted to a series of catastrophic changes for at least 500 years without a significant admixture from either European- or African-derived settlements adjacent to tribal communities [45].

In our study, no subject bore the rare *ε*2*ε*2 genotype which is concordant with previous studies in samples from Ecuadorian [16, 18], Mexican [46], Cuban [27], Colombian [36, 47], and Peruvian [43] populations. In addition, several studies suggest that the APOE *ε*2 allele is present at very low frequencies or absent in groups that populated the Americas in prehistoric times [44, 46, 48, 49]. De Andrade et al. (2000) proposed two possible explanations for these observations: (a) the APOE *ε*2 allele was present in the founding native American populations at very low frequencies and either was lost from some groups during the process of tribalization or was not identified due to restricted sample sizes; (b) the presence of the APOE *ε*2-allele is due to mixing with non-Indians [48].

Concerning the *ε*4*ε*4 genotype, it was only found within the case group. Correspondingly, a large meta-analysis has shown that the *ε*4*ε*4 genotype is found at very low frequencies in most healthy control populations worldwide: Caucasian (1.8%), African American (2.1%), Hispanic (1.9%), and Japanese (0.8%) [22].

We observed a higher frequency of the APOE *ε*4 allele in the LOAD cases compared to the healthy control subjects (33.9%/6.0% = 5.7). This coefficient is higher than the values reported worldwide in a large meta-analysis in Caucasian (36.7%/13.6% = 2.7), African American (32.2%/19.0% = 1.7), Hispanic (19.2%/11.0% = 1.7), and Japanese (27.8%/8.9% = 3.1) populations [22]. Furthermore, it is higher than those previously described in other Latin American populations as is shown in Table 4. The low APOE *ε*4 allele frequency in our control group could be because of the high LOAD risk it may be conferring in Ecuadorian population, making it less likely to be found in healthy individuals. It is worth mentioning that 12.0% of our control population had APOE genotypes carrying *ε*4 allele (*ε*3*ε*4, *ε*2*ε*4) and 50.0% of LOAD patients had APOE genotypes not carrying *ε*4 allele (*ε*3*ε*3, *ε*2*ε*3). This observation confirms that although the APOE *ε*4 allele is confirmed as a risk factor for AD, it is neither necessary nor sufficient to develop the disease, so there are probably other factors acting [42, 50, 51].

Genotype analyses revealed a significant association between APOE *ε*4 allele carriers and LOAD (OR = 7.286; 95% CI = 2.824–18.799; *p* < 0.001). These findings suggest that APOE *ε*4 indeed increases the risk of developing LOAD in the Ecuadorian Mestizo population, which is consistent with several studies [13, 22, 41]. The substantial risk for LOAD conferred by the APOE *ε*4 allele for Ecuadorians in this study contrasts with the nonexistent risk of LOAD previously reported [21]. Nevertheless, even though the previous study did not reveal a significant association between APOE *ε*4 allele carriers and LOAD, a noticeable difference between case and control groups was still observed in that study.

A low or nonexistent risk has been reported for “Hispanics” in worldwide studies where the majority of the individuals were of Caribbean origin [23], or of unspecified composition [22, 24]. However, when analyzing the risk per country in Latin Americans, it seems to be heterogeneous as is shown in Table 4. In Mexico, two studies have reported a lack of association between APOE *ε*4 allele carriers and LOAD, yet a small AD sample size (49 and 28 patients, resp.) should be mentioned as an important limitation in those studies [25, 26]. In Cuba, a first study including patients with probable AD diagnosis showed an increased risk for LOAD in *ε*4 carriers (OR = 4.33; IC = 1.27–14.79) [44]. Then, a second study confirmed that risk in a larger sample size; however, the patients included both probable and possible AD diagnosis and the association observed was lower (OR = 2.47; IC = 1.96–3.12) [28]. In Brazil, two independent studies, predominantly composed of individuals with European ethnic background, reported similar risks to each other for LOAD in *ε*4 carriers (OR = 3.63; IC = 2.19–6.03 and OR = 4.35; IC = 2.45–7.78) [24, 25]. Besides, the risk found in a small sample size (cases = 45, controls = 45) from Argentina was similar to those reported in Brazilians (OR = 3.33; IC = 1.20–9.02) [31]. Similarly, a moderate association has been reported in Venezuela [32, 33] and Chile [34]. In Colombia, the risks found are higher than those reported in the other Latin American countries [35, 36]. Particularly, the risk observed in our study between APOE *ε*4 allele carriers and LOAD is similar to that found in the Colombian study with patients diagnosed with probable AD (*ε*4 carriers: OR = 7.4; 95% CI = 2.5–22.0) [36]. This similarity may be due to the fact that the ancestry proportions of both countries range from predominantly Amerindian to predominantly European, with generally low levels of African descent [52].

Interestingly, the highest-risk associations between having one APOE *ε*4 allele and LOAD described in Table 4 are those reported in patients with a diagnosis of probable AD as our study. This observation suggests a higher frequency of *ε*4 in patients with a more accurate clinical diagnosis. The reasons could be either a subgroup of patients diagnosed with probable AD who could have an additional risk factor or another subgroup of patients diagnosed with possible AD who might not have the disease. Thus, the differentiation of this category should be taken into account when assessing the risk of APOE *ε*4 allele for LOAD.

Importantly, it should be considered that Hispanics are a mixture of European, African, and Amerindian genomes, with percentages depending on the country of origin [52, 53]. However, the term “Hispanic” is commonly incorrectly used because it tends to define any individual from the Caribbean, Central, and South America [53]. The ethnic diversity of the Hispanic subgroups is a consequence of their history. In the islands of the Caribbean, the native population was virtually extinguished after the coming of the Europeans. In the continent, two different native American empires, Mayas and Incas, were mainly concentrated in what now are Central America and Western South America, respectively [54]. At the beginning of the 16th century, the Inca Empire included today's Ecuador, Peru, Bolivia, and a large part of Chile, as well as smaller territories in Argentina and Colombia [55]. Currently, the Mestizo populations with the highest native ancestry are those located in areas which historically have had relatively large native populations [56]. Thus, the Ecuadorian Mestizo population has one of the largest Amerindian gene contributions among Hispanics [20]. Nevertheless, data often represents the Hispanic group as a whole ignoring the mentioned variability across the many subgroups, so it is possible that the differences in the ethnic composition of the Hispanic subgroups and the diversity within native American ancestors would be at least partially responsible for the different findings among Latin Americans. Altogether, these clearly indicate that a distinction across the Hispanic subgroups must be made when evaluating the implications of APOE *ε*4 allele risk for LOAD. Also, there may be interactions between APOE and other genes, ethnic groups, and environmental factors that might alter the risk for AD conferred by the APOE *ε*4 allele in different populations [8].

The relatively small size of the researched population should be mentioned with respect to our results as a limitation. Therefore, in order to overcome this limitation, patients were clinically diagnosed by a neurologist with probable AD using strict inclusion criteria according to NIA-AA and evidence of progressive cognitive decline so as to increase the level of clinical diagnosis certainty [37]. In addition, a careful selection of healthy controls was made through a neurological examination which discarded psychiatric and neurological impairments, and individuals with an MMSE score below 27 were excluded.

## 5. Conclusion

In conclusion, our results suggest that the APOE *ε*4 allele is a strong risk factor for LOAD in the Ecuadorian Mestizo population. However, further research with a larger sample size is needed in order to confirm the level of risk and to determine the APOE *ε*4 allele dose effect. Furthermore, we recommend studying the relationship between APOE *ε*4 allele and the rapid progress of LOAD, the APOE *ε*4 risk in other Ecuadorian ethnicities, and other genetic and environmental susceptibilities which could be regulating the predisposition to LOAD, as well as the conduction of prevalence and incidence AD studies on the Ecuadorian population. Finally, the control of different selected Hispanic subgroups should be taken into consideration when evaluating genetic contributions to LOAD risk, as these could directly affect the results.

## Figures and Tables

**Table 1:** Demographic and clinical characteristics.

	LOAD cases *n* = 56	Controls *n* = 58	*p*
Gender, female (%)	30 (53.6)	31 (53.4)	1.00^a NS^
Age ± SD, years	78.04 ± 6.32	76.62 ± 7.07	0.263^NS^
Smoking, yes (%)	18 (32.1)	13 (22.4)	0.309^a NS^
Hypertension, yes (%)	27 (48.2)	33 (56.9)	0.459^a NS^
Diabetes, yes (%)	8 (14.3)	7 (12.1)	0.942^a NS^
MMSE ± SD, score	18.07 ± 5.24	28.28 ± 1.25	<0.001^*∗*^
Education ± SD, years	10.27 ± 4.83	10.38 ± 4.24	0.896^NS^

^a^Corrected by Yates; NS: nonsignificant; ^*∗*^significant; SD: standard deviation; MMSE: Mini-Mental State Examination.

**Table 2:** APOE allele and genotype frequencies in case and control subjects.

Genotypes	LOAD cases (%) *n* = 56	Controls (%) *n* = 58
*ε*3*ε*3	27 (48.2)	47 (81.0)
*ε*3*ε*4	17 (30.4)	6 (10.3)
*ε*4*ε*4	10 (17.9)	0 (0.0)
*ε*2*ε*4	1 (1.8)	1 (1.7)
*ε*2*ε*3	1 (1.8)	4 (6.9)

Alleles	LOAD cases (%) *n* = 112	Controls (%) *n* = 116

*ε*3	72 (64.3)	104 (89.7)
*ε*4	38 (33.9)	7 (6.0)
*ε*2	2 (1.8)	5 (4.3)

**Table 3:** Risk association between APOE and LOAD.

Genotypes	*p*	OR	95% CI
Non-*ε*4 carriers		1^R^	
*ε*4 carriers	<0.001^*∗*^	7.286^*∗*^	2.824–18.799

^R^Reference; ^*∗*^significant; OR: odds ratio; CI: confidence interval.

**Table 4:** Studies on APOE and AD in the Latin American population.

Population	Cases/controls (*n*)	AD diagnosis	OR (95% CI)	*ε*4 frequencies (%) (cases/controls)
“Hispanics” [22]	261/267	Probable or definite	*ε*3*ε*4: 2.2 (1.3–3.4) *ε*4*ε*4: 2.2 (0.7–6.7) = n.s.	19.2/11.0 = 1.7

“Hispanics” Caribbean [23]	145/516	Unspecified	*ε*4 carriers: 1.1 (0.7–1.6) = n.s.	14.8/14.1 = 1.0

“Hispanics” [24]	140/219	Unspecified	APOE *ε*4 heterozygous: 1.6 (1.1–2.3) APOE *ε*4 homozygous: 2.5 (1.1–5.7)	19.0/10.0 = 1.9

Mexico [25]	28/28	Probable and possible	*ε*4 carriers: 2.04 (0.59, 7.10) = n.s.	21.4/12.5 = 1.7

Mexico [26]	49/141	Unspecified	*ε*4 carriers: 1.01 (0.45–2.23) = n.s.	13.2/10.9 = 1.2

Cuba [27]	80/21	Probable	*ε*4 carriers: 4.33 (1.27–14.79)	25.0/7.1 = 3.6

Cuba [28]	273/2247	Any dementia diagnosis	*ε*3*ε*4: 2.59 (2.04–3.28) *ε*4*ε*4: 2.88 (1.58–5.27) *ε*4 carriers: 2.47 (1.96–3.12)	17.4/8.0 = 2.2

Brazil [29]	104/108	Unspecified	*ε*4 carriers: 3.63 (2.19–6.03)	33.1/12.0 = 2.8

Brazil [30]	120/120	Probable	*ε*3*ε*4: 3.22 (1.73–6.01) *ε*4*ε*4: 9.57 (2.07–44.13) *ε*4 carriers: 4.35 (2.45–7.78)	31.0/10.0 = 3.1

Argentina [31]	45/45	Probable	*ε*4 carriers: 3.33 (1.20–9.02).	22.0/7.7 = 2.9

Venezuela [32]	121/1665	Unspecified	*ε*4 carriers: 2.97 (1.87–4.71)	17.0/11.0 = 1.5

Venezuela [33]	79/100	Unspecified	*ε*3*ε*4: 1.93 (1.02–3.70) *ε*4*ε*4: 4.29 (1.32–13.90)	34.8/16.0 = 2.2

Chile [34]	95/187	Probable and possible	*ε*3*ε*4: 2.4 (1.3–4.5) *ε*4*ε*4: 12.8 (3.9–47.6) *ε*4 carriers: 2.9 (1.7–5.1)	40.0/19.0 = 2.1

Colombia [35]	181/181	Unspecified	APOE *ε*4 heterozygous: 5.51 (3.32–9.16) APOE *ε*4 homozygous: 21 (2.79–6.48)	58.0/13.0 = 4.5

Colombia [36]	83/44	Probable and possible	*ε*4 carriers: 5.1 (1.9–13.6)	23.4/6.8 = 3.4

Colombia [36]	39/44	Probable	*ε*4 carriers: 7.4 (2.5–22.0)	30.8/6.8 = 4.5

Ecuador [21]	39/39	Unspecified	*ε*3*ε*4: 2.15 (0.6–7.9) = n.s. *ε*4*ε*4: 2.58 (0.2–76.8) = n.s.	17.9/10.3 = 1.7

Ecuador (current study)	56/58	Probable	*ε*4 carriers: 7.286 (2.824–18.799)	33.9/6.0 = 5.7

n.s.: no significant effect.
